# Human adenovirus infection induces pulmonary inflammatory damage by triggering noncanonical inflammasomes activation and macrophage pyroptosis

**DOI:** 10.3389/fimmu.2023.1169968

**Published:** 2023-04-18

**Authors:** Lexi Li, Huifeng Fan, Jinyu Zhou, Xuehua Xu, Diyuan Yang, Minhao Wu, Can Cao, Gen Lu

**Affiliations:** ^1^ School of Medicine, South China University of Technology, Guangzhou, China; ^2^ Department of Respiration, Guangzhou Women and Children’s Medical Centre, Guangzhou, China; ^3^ Department of Immunology, Zhongshan School of Medicine, Sun Yat-sen University, Guangzhou, China

**Keywords:** human adenovirus, inflammasome, caspase-4, caspase-5, pyroptosis

## Abstract

**Introduction:**

Human adenovirus (HAdV) is a common respiratory virus, which can lead to severe pneumonia in children and immunocompromised persons, and canonical inflammasomes are reported to be involved in anti-HAdV defense. However, whether HAdV induced noncanonical inflammasome activation has not been explored. This study aims to explore the broad roles of noncanonical inflammasomes during HAdV infection to investigate the regulatory mechanism of HAdV-induced pulmonary inflammatory damage.

**Methods:**

We mined available data on GEO database and collected clinical samples from adenovirus pneumonia pediatric patients to investigate the expression of noncanonical inflammasome and its clinical relevance. An *in vitro* cell model was employed to investigate the roles of noncanonical inflammasomes in macrophages in response to HAdV infection.

**Results:**

Bioinformatics analysis showed that inflammasome-related genes, including caspase-4 and caspase-5, were enriched in adenovirus pneumonia. Moreover, caspase-4 and caspase-5 expression levels were significantly increased in the cells isolated from peripheral blood and broncho-alveolar lavage fluid (BALF) of pediatric patients with adenovirus pneumonia, and positively correlated with clinical parameters of inflammatory damage. *In vitro* experiments revealed that HAdV infection promoted caspase-4/5 expression, activation and pyroptosis in differentiated THP-1 (dTHP-1) human macrophages via NF-κB, rather than STING signaling pathway. Interestingly, silencing of caspase-4 and caspase-5 in dTHP-1 cells suppressed HAdV-induced noncanonical inflammasome activation and macrophage pyroptosis, and dramatically decreased the HAdV titer in cell supernatants, by influencing virus release rather than other stages of virus life cycle.

**Discussion:**

In conclusion, our study demonstrated that HAdV infection induced macrophage pyroptosis by triggering noncanonical inflammasome activation via a NF-kB-dependent manner, which may explore new perspectives on the pathogenesis of HAdV-induced inflammatory damage. And high expression levels of caspase-4 and caspase-5 may be a biomarker for predicting the severity of adenovirus pneumonia.

## Introduction

Human adenovirus (HAdV), a member of the family Adenoviridae, is a common pathogen of respiratory tract infection in childhood and immunocompromised persons with high morbidity and mortality (1–3). There are seven different HAdV species (A-G), and to date, over 110 genotypes have been identified (1). And HAdV infection in children can cause numerous diseases such as pleural effusions, acute respiratory distress syndrome (ARDS), respiratory failure, myocarditis, and even death (3–6). Epidemiology suggests that among these serotypes, HAdV-3 and -7 are the most common types causing severe respiratory disease in children less than 5 years old worldwide (1–3, 7).

Inflammasomes, a group of cytosolic protein complexes, are formed to mediate host innate immune responses to microbial infection and cellular damage. They recruit inflammatory caspases, cysteine proteases that initiate or execute cellular programs, to trigger inflammation or cell death. Inflammasomes include canonical and noncanonical inflammasomes. Canonical inflammasomes, such as NLRP3, NLRP1, IPAF, and AIM2 inflammasome, activate caspase-1 to cleave pro-interleukin-1 beta (pro-IL-1β) and IL-18 into the secreted bioactive cytokines (8).. However, noncanonical inflammasomes often respond to intracellular lipopolysaccharide (LPS) and activate caspase-4 and caspase-5 in humans and caspase-11 in mice (9). Activated caspase-1/4/5/11 can induce cleavage of the pore-forming protein gasdermin D (GSDMD), leading to an inflammatory lytic type of cell death called pyroptosis.

It has been reported that HAdV DNA can activate inflammasomes to trigger innate immune responses (10). And K^+^ efflux, reactive oxygen species (ROS) and lysosomal damages have been brought forward as the exact cellular events involved in HAdV-induced NLRP3 inflammasome activation (11–13). Another study has shown that AIM2 inflammasome activated during HAdV infection to trigger caspase-1-mediated IL-1β/18 processing and GSDMD cleavage (14).

It is commonly believed that noncanonical caspase-4/5/11 directly senses cytosolic LPS *via* their caspase-activating and recruitment domains (CARD), pointing out the importance of noncanonical inflammasomes in anti-bacterial defense (15–17). Recently studies has verified that virus infection induces noncanonical inflammasomes activation during inflammatory responses, including severe acute respiratory syndrome coronavirus 2 (SARS–CoV-2), murine gammaherpesvirus 68 (MHV68) and coxsackievirus B3 (CVB3) (18–20). However, whether HAdV infection induced noncanonical inflammasomes activation in adenovirus pneumonia has not been explored.

Several signaling pathways play an important role in regulating inflammasome activation. For example, the nuclear factor-κB (NF-κB) signaling pathway serves as a prototypical proinflammatory pathway and provides the first signal for NLRP3 inflammasome activation by inducing pro-IL-1β and NLRP3 expression (21). NF-κB activation also promotes noncanonical caspase expression in inflammatory diseases (22, 23). Moreover, cGAS/STING signaling pathway functions as a cytoplasmic DNA sensor to initiate innate immune response against pathogen infection. The second messenger cGAMP catalyzed by cGAS could activate STING to induce downstream activation of TBK1/IRF3 or NF-κB, which respectively results in the production of type I interferons (IFNs) or proinflammatory cytokines (24–26). STING signaling pathway also contributes to both canonical and noncanonical inflammasome activation in *Chlamydia trachomatis* mouse bone marrow derived macrophages (BMDMs) (27). However, whether STING or NF-κB signaling pathway influences noncanonical inflammasome activation during HAdV infection remains unclear.

In the present study, we explored the role of noncanonical inflammasome during HAdV infection. We found that caspase-4 and caspase-5 expression levels were significantly increased in pediatric patients with adenovirus pneumonia and positively correlated with inflammatory damage. And *in vitro* experiments indicated that HAdV infection induced noncanonical inflammasomes activation and macrophage pyroptosis *via* NF-κB signaling pathway, while the STING signaling pathway was not involved in. Interestingly, silencing of caspase-4 and caspase-5 in dTHP-1 cells dramatically decreased the HAdV titer in cell supernatant. Overall, our study explored the broad role of noncanonical inflammasomes in HAdV-induced inflammatory responses, which may provide a potential therapeutic target for pediatric adenovirus pneumonia and a predictive biomarker for the severity.

## Material and methods

### Materials and reagents

RPMI medium, fetal bone serum (FBS), penicillin-streptomycin, L-glutamine, Opti-MEM and Lipofectamine™ 2000 were products of Invitrogen (Carlsbad, CA, USA). LPS derived from *Pseudomonas aeruginosa*, propidium iodide (PI), PMA and DMSO were purchased from Sigma Aldrich (St. Louis, MO, USA). C-176 (STING inhibitor) was bought from Selleck (Houston, TX, USA) and BAY11-7082 (NF-κB inhibitor) was bought from MedChemExpress (New Jersey, USA). 2’, 3’-cGAMP (STING agonist) and CpG ODN 2006 were bought from Invivogen (San Diego, CA, USA). Primary antibodies: anti-caspase-1 (AG-20B-0048) from Adipogen (San Diego, CA, USA); anti-caspase-4 (4450S), anti-GSDMD (97558S), anti-NF-κB p65 (8242S) and anti-P-NF-κB p65 (3033S) from Cell Signaling Technology (Beverly, MA, USA); another anti-caspase-4 (ab22687) from Abcam (Cambridge, MA, USA); anti-caspase-5 (M060-3) from Medical & Biological Laboratories(Nagoya, Japan); anti-β-actin (A1978) and anti-GAPDH(G9295) from Sigma Aldrich (St Louis, MO, USA).

### Bioinformatics analysis

We downloaded the series GSE103119 on the Gene Expression Omnibus (GEO) database, whose microarray platform is GPL10558 (Illumina HumanHT-12 V4.0 expression beadchip). And 20 healthy controls and 80 viral pneumonia samples, including 9 adenovirus pneumonia samples, were used in the present study. Based on the annotation information in the platform, the probes were transformed into the corresponding gene symbols. Then the data were normalized using quantile normalization with the lumi package in R software (Version 4.2.1). Differentially expressed genes (DEGs) were estimated by using an online tool GEO2R (http://www.ncbi.nlm.nih.gov/geo/geo2r ) with the following condition: adjusted *p*-value < 0.05 and the absolute value of log_2_fold-change (log_2_FC)> 2. We then performed Kyoto Encyclopedia of Genes and Genomes (KEGG) pathway and Gene Ontology (GO) enrichment analysis for genes with these DEGs using Metascape (www.metascape.org ), which provides automated meta-analysis tools to reveal common and unique pathways from 40 independent knowledge bases. Gene Set Enrichment Analysis (GSEA) software (Version 4.3.0) was also used to explore the potential biological function difference between the two groups. GSEA was run for the “REACTOME_PYROPTOSIS.v2022.1.Hs.gmt” gene sets. Besides this, the correlations between variables were evaluated by a *Pearson* rank correlation test. The heatmaps and the correlation matrices were plotted by Chiplot (https://www.chiplot.online/ ), a free online platform for data analysis and visualization.

### Clinical specimens and data collection

A total of 28 adenovirus pneumonia pediatric patients were enrolled in the present study. They were diagnosed according to the evidence-based guidelines regarding the diagnosis of pneumonia in children published by the World Health Organization (28). And the evidence of HAdV was confirmed by positive multiplex polymerase chain reaction (PCR) from lower respiratory tract samples. 15 pediatric patients were selected as control subjects, who were verified without recent respiratory infection by clinical characteristics and image manifestations. Severe or non-severe pneumonia was classified on the basis of clinical features detailed as previously reported (29). Peripheral blood and/or broncho-alveolar lavage fluid (BALF) samples, as well as clinical examination data from all the participants, were collected. All the pediatric participants were recruited from Guangzhou Women and Children’s Medical Center (Guangzhou, China), and written informed consents were obtained from all the participants’ guardians. This study was conducted in accordance with the declaration of Helsinki and approved from the Ethics Committee of the School of Medicine in the South China University of Technology and the Ethics Committee of Guangzhou Women and Children’s Medical Center. Detailed clinical characteristics and laboratory information are shown in **Table 1**.

**Table 1:** Summary of clinical features and laboratory results of adenovirus pneumonia pediatric patients.

	Control cases (n=15)	Non-severe cases (n=12)	severe cases (n=16)	P value
**Age (years)**	4.01 ± 3.12	4.51 ± 2.27	2.81 ± 2.01	
**Gender (male/female)**	8/7	6/6	6/10	
**White blood cell counts (× 10⁹/L)**	6.83 ± 0.30	9.91 ± 1.14	16.07 ± 1.82	0.017
**Neutrophil (%)**	44.93 ± 2.70	43.25 ± 4.80	63.01 ± 3.00	0.002
**Neutrophil (× 10⁹/L)**	3.04 ± 0.22	5.14 ± 0.92	9.39 ± 1.44	0.032
**Monocytes count (× 10⁹/L)**	0.39 ± 0.02	0.87 ± 0.16	1.34 ± 0.22	0.122
**Lymphocyte count (× 10⁹/L)**	3.23 ± 0.25	3.16 ± 0.39	4.72 ± 0.65	0.075
**Platelet count (× 10⁹/L)**	304.27 ± 19.87	398.50 ± 28.34	400.07 ± 37.51	0.976
**Hemoglobin (g/L)**	114.36 ± 8.11	124.00 ± 2.74	115.20 ± 4.91	0.171
**Hypersensitive-C-reactive protein (mg/L)**	0.45 ± 0.47	17.70 ± 7.36	118.47 ± 22.50	0.007
**Procalcitonin (ng/mL)**		0.33 ± 0.11	2.45 ± 0.56	0.003
**Erythrocyte sedimentation rate (mm/h)**		16.80 ± 4.61	34.55 ± 9.24	0.257
**PH**		7.40 ± 0.02	7.38 ± 0.02	0.573
**PaO_2_(kPa)**		9.83 ± 2.40	8.58 ± 0.62	0.295
**PaCO_2_(kPa)**		4.94 ± 0.68	5.54 ± 0.49	0.451
**Sodium (mmol/L)**		136.23 ± 3.18	135.26 ± 0.79	0.524
**Potassium (mmol/L)**		3.59 ± 0.41	3.75 ± 0.15	0.550
**Lactic acid (mmol/L)**		1.51 ± 0.18	2.04 ± 0.23	0.156
**Alanine aminotransferase (U/L)**		16.00 ± 2.96	21.69 ± 8.78	0.670
**Aspartate aminotransferase (U/L)**		34.38 ± 3.37	35.88 ± 5.45	0.860
**Creatine kinase-MB (U/L)**		20.00 ± 2.45	17.38 ± 1.91	0.441
**Lactate dehydrogenase (U/L)**		231.50 ± 20.63	302.50 ± 20.08	0.033
**Creatinine (µmol/L)**		33.38 ± 5.78	34.13 ± 4.93	0.930
**Total bilirubin (µmol/L)**		3.71 ± 0.73	5.42 ± 0.62	0.123
**Direct bilirubin (µmol/L)**		1.38 ± 0.31	1.82 ± 0.23	0.285
**Albumin (g/L)**		0.006726994	34.61 ± 1.19	0.007
**Prothrombin time (s)**		0.025106203	14.39 ± 0.26	0.025
**Activated partial thromboplastin time (s)**		0.115970773	38.58 ± 1.34	0.116
**Fibrinogen (g/L)**		0.02422345	5.11 ± 0.45	0.024

### Cell culture and differentiation

Human THP-1 cells were cultured in RPMI medium supplemented with 10% FBS, 1% penicillin-streptomycin and 1% L-glutamine. And cells were incubated at 37°C in a humidified incubator with 5% CO_2_. The THP-1 cells were seeded in 12-well plates (8×10^5^ cells/well) or 24-well plates (4×10^5^ cells/well), and differentiated with PMA (100 nM) overnight. For mock differentiation, no PMA was used in the procedure.

### Cell stimulation and transient transfection

In some cases, differentiated THP-1 (dTHP-1) cells were pretreated with the specific inhibitors, such as C-176 (1μM) and BAY11-7082 (10nM) for 1h before the infection and transfection. According to the manual of Lipofectamine™ 2000, dTHP-1 cells were transiently transfected with specific small interfering RNA against caspase-4 and caspase-5 vs scrambled control siRNA (siNC) or siGSDMD vs siNC. All siRNAs were designed and synthesized by Ruibo Biotechnology (Guangzhou, Guangdong, China). Flagellin transfection was performed according to the manual of DOTAP Liposomal Transfect Reagent (Sigma Aldrich, St. Louis, MO, USA). To stimulate TLR9, its respective ligands CpG DNA (10μg/mL) was added directly into the culture media. To stimulate STING, cGAMP (10μg/mL) was delivered into cytoplasm of dTHP-1 cells by using Lipofectamine™ 2000.

### Virus infection and titration

The virus used in this study was the HAdV-3 strain GZ1 (GenBank accession number, DQ099432), generously provided by Dr. Qiwei Zhang at the Institute of Medical Microbiology, Jinan University. HAdV was propagated in A549 cells, with which the virus titers were determined using tissue culture infective dose (TCID_50_) assay. The virus was inoculated into dTHP-1 cells at a multiplicity of infection (MOI) of 100. The infection medium was removed 2h post adsorption and then cultured in fresh RPMI medium supplied with 10% FBS. The culture supernatants were sampled at indicated times to assess virus titer using TCID_50_.

### Virus binding, entry and replication assay

In the virus binding assay, the dTHP-1 cells were incubated with HAdV (MOI=100) in 4° for 1h as a previous study (30). In the virus entry assay, the dTHP-1 cells were incubated with HAdV (MOI=100) in 37° for 0.5h to allow for virus attachment and internalization. In the virus replication assay, the dTHP-1 cells were incubated with HAdV (MOI=100) in 37° for 2h. The infection medium was removed 2h post adsorption and then cultured in fresh RPMI medium supplied with 10% FBS. The supernatants of cells were discarded, followed by washing with PBS buffer for three times. Total cellular DNA was extracted from the infected cells using Hipure Tissue DNA Mini Kit (Magen, Guangzhou, China). Relative HAdV-3 DNA expression fold was quantified by real-time PCR as a previous study (31). HAdV primer sequence was provided in Supplementary **Table 1**.

### Western blot

Cells were lysed with cell lysis buffer containing 1mM phenylmethylsulfonyl fluoride, 1% protease inhibitor cocktail, 1% phosphorylase inhibitor cocktail, and 1 mM dithiothreitol (all from Sigma Aldrich, St. Louis, MO, USA). Cell lysate samples were boiled, separated on SDS-PAGE, and then transferred to PVDF membranes. Membranes were blocked with 5% (w/v) nonfat milk and incubated with a primary antibody overnight at 4°C, followed by a second incubation at room temperature for 1-2 h with appropriate HRP-conjugated secondary antibodies. After further washing with PBST, blots on the membranes were visualized with ECL reagent (KeyGEN, Nanjing, China) according to the manufacturer’s protocol. Equal protein loading was confirmed in all the experiments by using GAPDH or β-actin as loading control.

### Real-time PCR

Total RNA was isolated from cell pellets using TRIzol (Invitrogen, Carlsbad, CA, USA) according to the manufacturer’s instruction, and quantitated using a NanoDrop 2000C Spectrophotometers (Thermo Scientific, West Palm Beach, FL, USA). One μg of total RNA was reverse-transcribed to produce cDNA by using the Revert Aid First Strand cDNA synthesis kit (Thermo Fisher Scientific, Waltham, MA, USA). Then the cDNA was amplified using SYBR green master mix (TaKaRa, Mountain View, CA, USA) following the manufacturer’s protocol. Quantitative real-time PCR were performed using a Bio-Rad CFX96 Real-Time PCR System. Real-time PCR primers sequences are provided in Supplementary **Table 1**. Relative expression levels were calculated with the 2^−ΔCt^ method. Relative mRNA levels were calculated after normalization to the level of β-actin.

### Enzyme-linked immunosorbent assay

Secreted IL-1β level in culture supernatants was determined by human IL-1β ELISA kits from Dakewe Biotechnology (Shenzhen, Guangdong, China), according to the manufacturer’s instructions.

### Propidium iodide staining

The dTHP-1 cells were inoculated on a 24-well plate. Following the treatment, PI staining solution was added to each well (0.3μg/ml). Cells were incubated at room temperature for 10-15 min and then observed under a fluorescence microscope. Three non-overlapping fields were randomly taken and photographed with the inverted fluorescent microscope Leica DMI4000B. The proportion of PI^+^ cells was calculated as follows: proportion of PI^+^ cells (%) = (number of red fluorescent cells/total cells) × 100%. Two wells were set in each group, and the experiment was repeated three times. The fluorescence images were analyzed and processed using ImageJ software to calculate the relative fluorescence density.

### Statistical analysis

Statistical analysis was performed using GraphPad Prism (Version 8.0.2). Differences between the two groups were compared by using Student’s t-test. Differences with a *p* value less than 0.05 were considered statistically significant.

## Results

### Inflammasome-related genes and signaling pathways were enriched among children hospitalized with viral pneumonia

**Figure 1:**
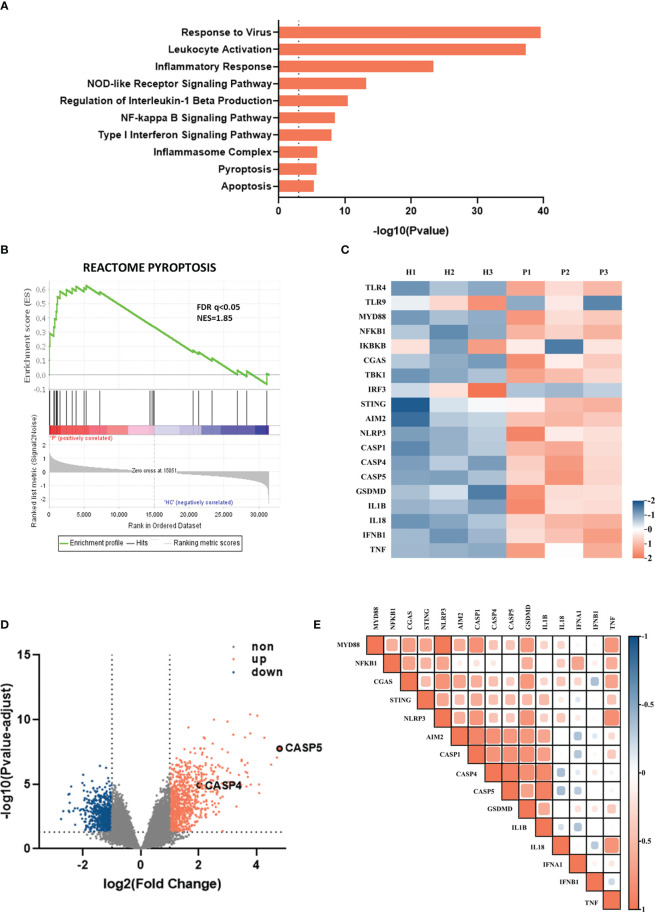
Inflammasome-related genes and signaling pathways were enriched among children hospitalized with viral pneumonia. The data, downloaded within the GSE103119 dataset on the GEO database, were normalized using quantile normalization with the lumi package in R. **(A)** Enrichment analysis showing some of significantly enriched signaling pathways in viral pneumonia pediatric patients compared to healthy controls. **(B)** GSEA enrichment plot of the pyroptosis pathway. **(C)** Heatmap depicting the expression profiling of inflammasome-related DEGs in adenovirus pneumonia pediatric patients compare to healthy controls. **(D)** Volcano plot of DEGs in adenovirus pneumonia children and healthy control. **(E)** Heatmap of pairwise correlation of inflammation-related genes expression with caspase-4 and caspase-5.

To unravel the immune regulatory mechanisms of viral pneumonia, we first analyzed changes in the gene transcriptome of whole blood from viral pneumonia pediatric patients and healthy controls by performing the enrichment analysis. The GO and KEGG analysis elucidated numerous statistically enriched biological terms and indicated that the most significantly changed gene enrichment pathways in viral pneumonia pediatric patients were the response to virus (GO:0009615) and the leukocyte activation (GO:0045321). Meanwhile, the pyroptosis (GO: 0070269) and the inflammasome complex pathway (GO: 0061702) were implicated as factors likely to be important for host responses to viral infection (*p*<0.001) (**Figure 1A**). The correlation between viral pneumonia and the pyroptosis signaling pathway was further confirmed by GSEA (**Figure 1B**). Furthermore, heatmaps indicated that the levels of inflammasome-related genes, such as NLRP3, AIM2, caspase-1/4/5, and inflammatory cytokines, including IL-1β/18, IFN-β, and tumor necrosis factor (TNF), were increased in adenovirus pneumonia pediatric patients (**Figure 1C**). Besides this, volcano plot analysis of adenovirus pneumonia pediatric patients also showed that caspase-4 and caspase-5 were remarkably upregulated compared to healthy controls (|log_2_FC|>2) (**Figure 1D**). As shown in the heatmap profile, IL-1β and GSDMD indicated positive correlation with caspase-4 and caspase-5 in adenovirus pneumonia pediatric patients, respectively (**Figure 1E**). Above all, we speculated that noncanonical caspase-4/5 inflammasome may be involved in modulating inflammatory responses during adenovirus pneumonia.

### Caspase-4 and caspase-5 expression levels were increased and positively correlated with inflammatory damage

**Figure 2:**
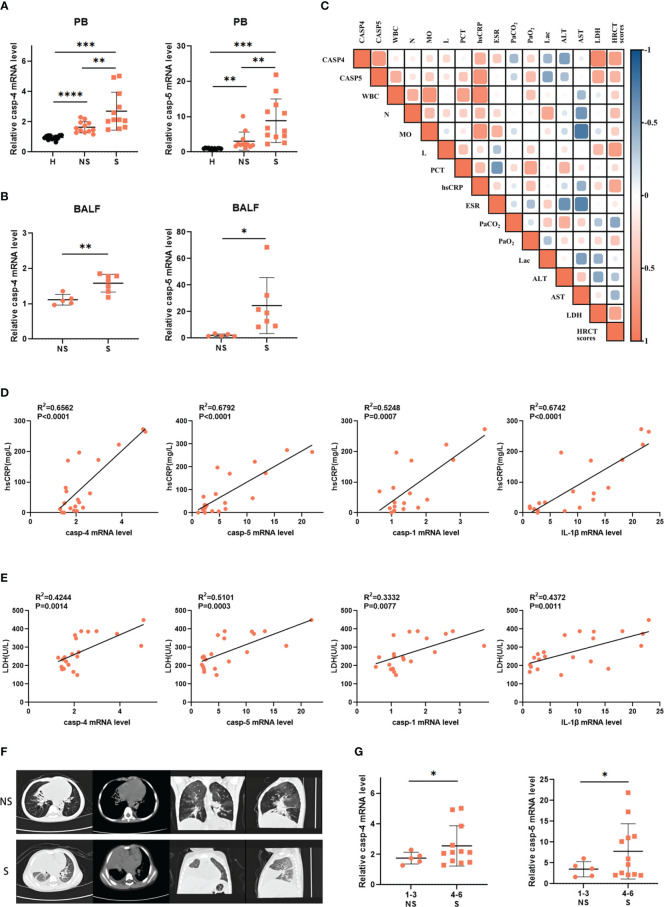
Caspase-4 and caspase-5 expression levels were increased and positively correlated with inflammatory damage. **(A)** Expression levels of caspase-4 and caspase-5 in PBMCs isolated from adenovirus pneumonia pediatric patients (12 non-severe vs 12 severe) and healthy volunteers’ (n=15) peripheral blood samples or **(B)** in cells isolated from severe (n=7) and non-severe patients (n=5) BALF samples. **(C)** Correlation matrix of caspase-4 and caspase-5 levels of pediatric patients (n=9) and their clinical parameters. **(D, E)** Correlations of indicated clinical inflammatory parameters of pediatric patients (n=20) with inflammation-related genes, respectively. **(F)** The representative lung HRCT images of severe and non-severe adenovirus pneumonia pediatric patients. **(G)** Expression levels of caspase-4/5 in adenovirus pneumonia pediatric patients (n=17) divided into two groups according to the HRCT scores. Data are representative of at least three experiments. Error bars represent the mean ± SEM. *P value<0.05, **P value <0.01, ***P value <0.001, ****P value <0.0001, ns, no significance.

To further verify these findings from transcriptome analysis, we collected clinical samples from adenovirus pneumonia pediatric patients, whose detailed clinical characteristics and laboratory information are shown in **Table 1**, to measure caspase-4 and caspase-5 expression by real-time PCR. As shown, peripheral blood mononuclear cells (PBMCs) from adenovirus pneumonia pediatric patients and healthy volunteers were enrolled, as well as cells of BALF samples from non-severe and severe pneumonia patients. We found that caspase-4 and caspase-5 mRNA levels significantly upregulated in the PBMCs of pneumonia patients with severe phenotype, while they slightly increased in non-severe patients compared to healthy controls, and increased in BALF of severe cases compared with the non-severe group (**Figures 2A, B**). Next, *Pearson* correlation matrix showed that caspase-4 and caspase-5 levels were positively associated with hypersensitive-C-reactive protein (hsCRP) and lactate dehydrogenase (LDH) concentration in peripheral blood (**Figure 2C**). As expected, the expression levels of caspase-1/4/5 and IL-1β in PBMCs of pediatric patients were founded to be positively correlated with hsCRP and LDH, respectively (**Figures 2D, E**).

Moreover, we also classified according to the high-resolution computed tomography (HRCT) scores ranging from 1 to 6 as a previous study (32), and divided all patients into two groups to evaluate whether caspase-4/5 expression was correlated with the degree of lung inflammatory damage (**Figure 2F**). It turned out that expression of caspase-4 and caspase-5 was higher in patients whose HRCT scores were 4-6 than in the 1-3 group (**Figure 2G**). Taken together, our results showed that caspase-4 and caspase-5 expression was increased in pediatric patients with adenovirus pneumonia and positively correlated with inflammatory damage. Thus, these data highlighted the potential role of noncanonical inflammasome in HAdV-induced inflammatory responses.

### HAdV infection induced activation of caspase-4 and caspase-5

**Figure 3:**
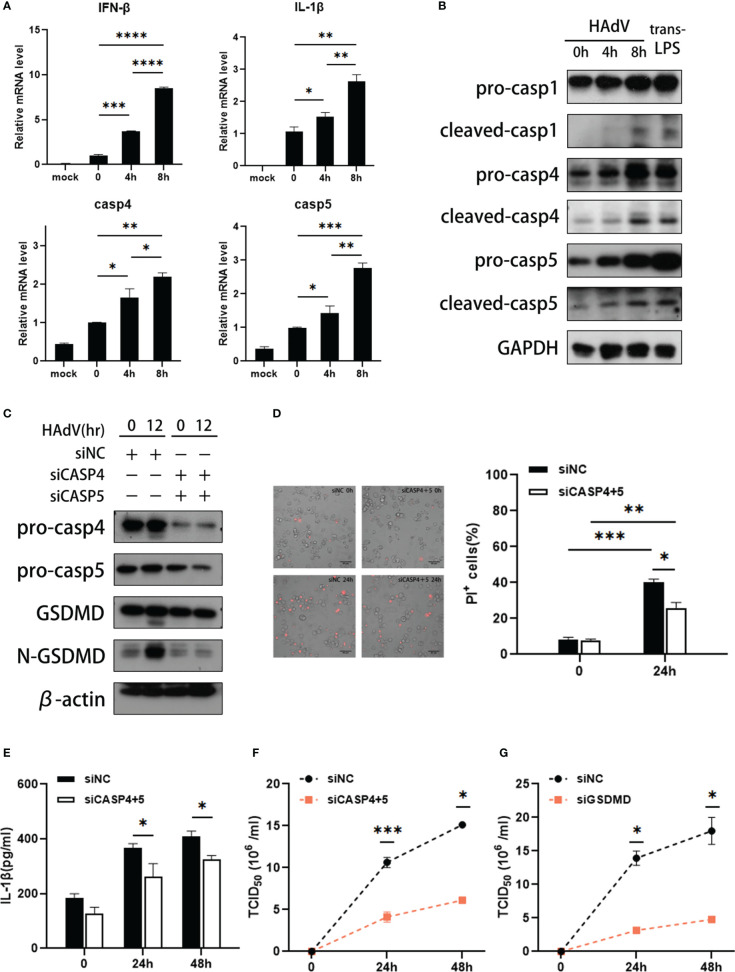
HAdV infection induced activation of caspase-4/5. The dTHP-1 cells were infected by HAdV (MOI=100) for indicated times and/or transfected LPS (2.5μg/ml) for 8h. **(A)**The mRNA levels of IFN-β, IL-1β, caspase-4/5 responses to HAdV infection at 0, 4, and 8h were measured by real-time PCR. **(B)** Western blot data showing expression of indicated protein in in dTHP-1 cells infected with HAdV at indicated times. **(C)** Western blot data showing expression of indicated protein in siCASP-4+5-treated dTHP-1 cells infected with HAdV at indicated times. **(D)** Representative images of dead cells stained with PI were observed by fluorescent microscopy (magnification: 20×, scale bar: 100 μm) and quantification of PI^+^ cells. **(E)** IL-1β secretion was tested by ELISA at 24h or 48h post-infection. The supernatants of **(F)** siCASP4+5 or **(G)** siGSDMD-treated vs siNC-treated dTHP-1 cells were collected for HAdV titer measured by the TCID_50_. Data are representative of two or three experiments. Error bars represent the mean ± SEM. *P value<0.05, **P value <0.01, ***P value <0.001, ****P value <0.0001.

Furthermore, we investigated the effects of HAdV infection on expression and activation of caspase-4 and caspase-5 *in vitro*. The dTHP-1 cells were infected with HAdV or transfected with LPS, a positive control inducing noncanonical inflammasome activation. Firstly, increased IFN-β and IL-1β mRNA levels indicated that HAdV successfully infected dTHP-1 cells (**Figure 3A**). The data also indicated that caspase-4 and caspase-5 mRNA levels were greatly elevated after 4h post-infection (**Figure 3A**). Western blot data exhibited that HAdV infection induced activation of pro-caspase-1/4/5, as reflected by the appearance of their p20 subunits in a time-dependent manner (**Figure 3B**). Given caspase-4 and caspase-5 underwent processing, noncanonical inflammasomes seemed to be activated in human macrophages infected with HAdV.

To ascertain the individual roles of noncanonical inflammasomes in HAdV-induced macrophage pyroptosis, we transfected dTHP-1 cells with siRNAs targeting the expression of corresponding caspases or scrambled control siRNA prior to HAdV infection. Compared to the control, caspase-4 and caspase-5 siRNA duplexes effectively suppressed caspase-4 and caspase-5 expression (**Figure 3C**). And HAdV infection strongly induced the cleavage of GSDMD, which could be attenuated by silencing of caspase-4 and caspase-5 (**Figure 3C**). We also observed the fraction of PI^+^ cells was decreased by silencing of caspase-4 and caspase-5 (**Figure 3D**). And ELISA data showed that silencing of caspase-4 and caspase-5 decreased secretion of IL-1β, implying that HAdV-induced noncanonical inflammasome activated to promote IL-1β release (**Figure 3E**). Collectively, caspase-4 and caspase-5 can induce pyroptosis in dTHP-1 cells during HAdV infection.

To identify whether the noncanonical inflammation has any effects on HAdV infection, the supernatants of dTHP-1 cells infected at different timepoints were harvested. The TCID_50_ assay data showed that silencing of caspase-4 and caspase-5 in dTHP-1 cells decreased the virus titer of HAdV when compared to the control group (**Figure 3F**). As reported, the HAdV life cycle includes five main phases, which are binding, entry, replication, assembly, and release (33, 34). To explore which stage is affected by noncanonical inflammasomes, we constructed HAdV binding and entry models. Real-time PCR data showed that silencing of caspase-4 and caspase-5 did not influence the amount of HAdV bound on the cell surface or entered in the cell (Supplementary **Figures 2A, B**). Furthermore, intracellular HAdV DNA level in siCASP4+5 vs siNC-transfected and siGSDMD vs siNC-transfected dTHP-1 cells are comparable at different timepoints post-infection (Supplementary **Figures 2C, D**), indicating that silencing of caspase-4/5 and GSDMD did not affect the HAdV replication. Meanwhile, silencing of GSDMD significantly decreased the HAdV titer in cell culture supernatants compared with the control group (**Figure 3G**). Based on these results, we speculated that noncanonical caspase-4/5 inflammasome activation and macrophage pyroptosis may promote HAdV release, rather than other virus cycle stages.

### NF-κB signal pathway was involved in HAdV infection-induced noncanonical inflammasome activation and macrophage pyroptosis

**Figure 4:**
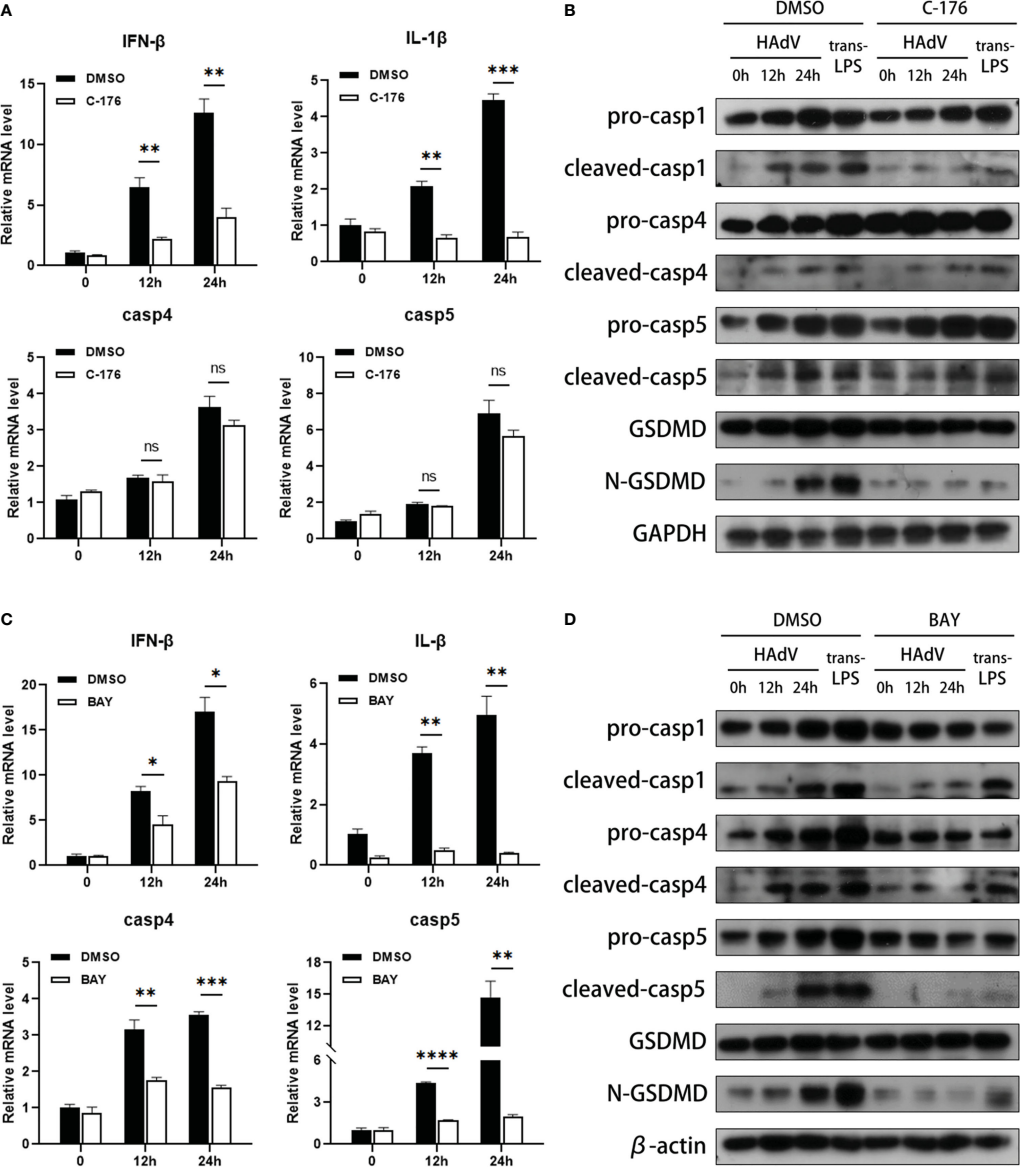
NF-κB was involved in noncanonical inflammasome activation and macrophage pyroptosis. The dTHP-1 cells pre-treated by either **(A, B)** C-176 (1μM) or **(C, D)** BAY11-7082 (10nM) vs vehicle control (DMSO) were infected with HAdV (MOI=100) for the indicated times or transfected with LPS (2.5μg/ml) for 8h. **(A, C)** The mRNA levels of IFN-β, IL-1β, and caspase-4/5 were measured by real-time PCR. **(B, D)** Protein levels of indicated molecular in cell lysates were tested by western blot. Data are representative of at least three experiments. Error bars represent the mean ± SEM. *P value<0.05, **P value <0.01, ***P value <0.001, ****P value <0.0001, ns, no significance.

We next explored the downstream molecules of HAdV infection in modulating caspase-4/5 activation and subsequent macrophage pyroptosis. Given that STING and NF-κB signaling pathway are reported to be involved in inflammasome activation, we used the C-176 (STING inhibitor) or BAY11-7082 (NF-κB inhibitor) to investigate the effects of STING and NF-κB signaling pathways on noncanonical inflammasome activation during HAdV infection. Notably, pyroptosis usually occurred within 12-24h after injury (35, 36), so the duration of infection extended to 12-24h after being pre-treated with indicated inhibitors. Pretreatment of STING inhibitor C-176 dramatically decreased the IFN-β and IL-1β mRNA expression, but slightly reduced NF-κB p65 phosphorylation (**Figure 4A** and Supplementary **Figure 1A**). Western blot data showed that C-176 pretreatment also suppressed the cleavages of caspase-1 and GSDMD at indicated times after HAdV infection or LPS transfection (**Figure 4B**), indicating that inhibition of STING attenuated canonical inflammasome activation and macrophage pyroptosis. Notably, no significant differences in the mRNA and protein levels of caspase-4/5 were observed in the dTHP-1 cells pre-treated with C-176 inhibitor and DMSO, suggesting STING signaling pathway was not involved in noncanonical inflammasome activation during HAdV infection (**Figures 4A, B**). Real-time PCR data also indicated that mRNA levels of caspase-4 and caspase-5, as well as IFN-β and IL-1β, were downregulated in BAY pre-treated group (**Figure 4C**). Additionally, western blot further confirmed that BAY pretreatment significantly decreased the expression and cleavage of caspase-4/5, as well as their downstream GSDMD (**Figure 4D**). To investigate whether NF-κB-mediated caspase-4/5 upregulation *via* TLR9 or STING, dTHP-1 cells were transfected with cGAMP (STING agonist) or stimulated with CpG ODN 2006 (TLR9 agonist). Real-time PCR data showed that CpG ODN 2006 increased the mRNA levels of caspase-4 and caspase-5, while cGAMP had no significant effect on their expression (Supplementary **Figures 1B, C**). In conclusion, NF-κB, but not STING signaling pathway, may be involved in HAdV-induced noncanonical inflammasome activation and macrophage pyroptosis.

## Discussion

Clinical observations have suggested that disease severity and outcomes of HAdV infection are closely associated with release of proinflammatory cytokines (37). Inflammasome-mediated inflammatory responses have been implicated in various microbial infections. However, only canonical inflammasomes, such as NLRP3 and AIM2 inflammasome, have been reported to be activated during HAdV infection (11–14). Here, we utilized wild-type HAdV-3 for the first time, which is one of the most common types causing severe adenovirus pneumonia in children, to investigate whether HAdV infection induces noncanonical inflammasome activation.

We mined available data on GEO database and found that expression of caspase-4 and caspase-5 was elevated in adenovirus pneumonia pediatric patients. Similarly, caspase-4 and caspase-5 expression levels were increased in the cells isolated from peripheral blood plasma and BALF, which collected from adenovirus pneumonia pediatric patients, and positively correlated with the severity of adenovirus pneumonia. HAdV exhibits species-restricted phenotypes, making studying disease progress in animal models particularly problematic (38). Thus, the mechanism and function of noncanonical inflammasomes during HAdV infection were investigated by *in vitro* cell model. Since macrophage is the major immune cell type for inflammasome activation, we stimulated human monocyte cell line THP-1 with PMA, to differentiate them into macrophages (dTHP-1) as previously reported (10–12). In our study, cell experiments identified the intracellular activation of caspase-4 and caspase-5 induced by HAdV infection. Furthermore, treatment with the caspase-4 and caspase-5 siRNAs attenuated HAdV-induced cleavage of GSDMD, confirming that HAdV infection induced macrophage pyroptosis by activating noncanonical inflammasomes.

Pyroptosis is a key function of canonical and noncanonical inflammasomes and usually plays essential roles in eliminating pathogenic infections (39). However, it is still unclear what influences noncanonical inflammasomes have on virus infection. It is reported that caspase-1 silencing enhances chikungunya virus (CHIKV) replication but severely impairs epstein-barr virus (EBV) replication (40, 41). Moreover, other studies find that the absence of canonical and noncanonical inflammasomes has no effect on replication of some viruses (18, 19, 40). The HAdV life cycle includes binding, entry, replication, assembly, and release (33, 34). We found that silencing of caspase-4 and caspase-5 did not affect the HAdV binding, entry, and replication. Meanwhile, silencing of caspase-4/5 and GSDMD in dTHP-1 cells decreased the virus titer of HAdV in cell culture supernatants, respectively. When viral particles are produced and accumulated in the infected cells, canonical and noncanonical inflammasome-induced macrophage pyroptosis will facilitate the viral progeny release (42). In this regard, we speculated that silencing of caspase-4 and caspase-5 suppressed noncanonical inflammasome-induced macrophage pyroptosis, and therefore impeded HAdV release. The effects of noncanonical inflammasomes-mediated pyroptosis have on virus load, inflammatory responses and lung injury would be better to be further investigated *in vivo* once HAdV-infected animal model has been validated.

We next explored the downstream molecules of HAdV infection in modulating caspase-4/5-mediated macrophage pyroptosis. In order to investigate if STING and NF-κB signaling pathway regulate noncanonical inflammasomes activation during HAdV infection, we pretreated dTHP-1 cells with NF-κB inhibitor before infected or transfected. Our results showed that cleavages of caspase-1 and GSDMD were suppressed in STING inhibitor group, confirming that STING signaling pathway was involved in canonical inflammasome activation. But STING inhibitor had no effect on caspase-4 and caspase-5 expression and cleavages, implying that STING signaling pathway was not involved in noncanonical inflammasome activation. We also found that NF-κB inhibitor suppressed HAdV-induced caspase-4 and caspase-5 expression, as well as decreased the cleavages of caspase-4/5 and downstream GSDMD. It is reported that during HAdV infection, both TLR9 and STING can recognize viral DNA and induce NF-κB activation (11, 12, 14). Whereas our *in vitro* data showed that activation of TLR9, but not STING, by using their specific agonists enhanced caspase-4 and caspase-5 expression. Therefore, we speculated that HAdV infection induced caspase-4/5 expression mainly *via* TLR9, rather than STING. Taken together, our current findings indicated that NF-κB, but not STING signaling pathway, may be involved in HAdV-induced noncanonical inflammasome activation and thereafter macrophage pyroptosis.

Canonical and noncanonical inflammasome both can trigger corresponding caspase activation to induce pyroptosis. In this regard, they act independently and in parallel to each other. Notably, they work in concert to protect the host against intracellular pathogens, but the interaction remains unclear. It is reported that activated noncanonical caspases, including caspase-4/5/11, can induce K^+^ efflux and then activate canonical NLRP3 inflammasome in some cases (43). And canonical inflammasomes can also act upstream of noncanonical inflammasomes in the host. As another study has reported, caspase-1 induces production of IL-18, which then triggers IFN-γ to prime caspase-11-mediated inflammatory responses during *B. thailandensis* infection (44). In addition, Akhter et al. found that caspase-11 was dispensable for caspase-1 activation in response to *Legionella, Salmonella, Francisella* and *Listeria* (45). Given that more attention has focused on individual impacts that noncanonical inflammasomes have, their interactive and collaborative contributions between canonical and noncanonical inflammasomes were less studied in our present study. Further studies on whether and how they cooperate to defend against HAdV are needed in the future.

In conclusion, our study provided evidence for the first time that HAdV infection induced caspase-4 and caspase-5 activation and thereafter macrophages pyroptosis. And silencing of caspase-4 and caspase-5 in dTHP-1 cells dramatically decreased the HAdV titer in cell supernatants, by influencing virus release. These findings explore new perspectives on the pathogenesis of HAdV-induced inflammatory damage. Moreover, expression of caspase-4 and caspase-5 were increased in pediatric patients with adenovirus pneumonia and positively correlated with severity and other clinical inflammatory parameters. Thus, high expression levels of caspase-4 and caspase-5 may be a biomarker for predicting the severity of adenovirus pneumonia.

## Data availability statement

The original contributions presented in the study are included in the article/**Supplementary Material**. Further inquiries can be directed to the corresponding authors.

## Ethics statement

The studies involving human participants were reviewed and approved by the Ethics Committee of the School of Medicine in the South China University of Technology the Ethics Committee of Guangzhou Women and Children’s Medical Center. Written informed consent to participate in this study was provided by the participants’ legal guardian/next of kin.

## Author contributions

LL, CC and JZ performed the experiments and bioinformatics analyses. HF and XX collected specimens and clinical data of pediatric patients and classified them with HRCT score. GL and DY performed bronchoscopy with BAL. CC, MW, and HF conceived the study. LL and CC drafted the manuscript. GL and MW performed critical revision. All authors contributed to the article and approved the submitted version.
