# Supplementary material for: Human adenovirus infection induces pulmonary inflammatory damage by triggering noncanonical inflammasomes activation and macrophage pyroptosis
Source: Front Immunol. 2023 Apr 18;14:1169968. doi: 10.3389/fimmu.2023.1169968 (PMC10167768; doi:10.3389/fimmu.2023.1169968)
Supplement: Supplementary file 1 [file Image_1.pdf]

Supplementary Image

Human Adenovirus Infection Induces Pulmonary Inflammatory Damage by Triggering Noncanonical Inflammasomes Activation and Macrophage Pyroptosis

Lexi Li[1, 2†], Huifeng Fan[2†], Jinyu Zhou[3], Xuehua Xu[2], Diyuan Yang[2], Minhao Wu[3], Can Cao[3*], Gen Lu[2, 1*]

[†]The authors contributed equally to this work.

*** Correspondence:**

Gen Lu, M.D., Ph.D.

E-mail: lugen5663330@sina.com

Can Cao.

E-mail: caoc9@mail.sysu.edu.cn

Minhao Wu, Ph.D.

E-mail: wuminhao@mail.sysu.edu.cn

Supplementary Figures

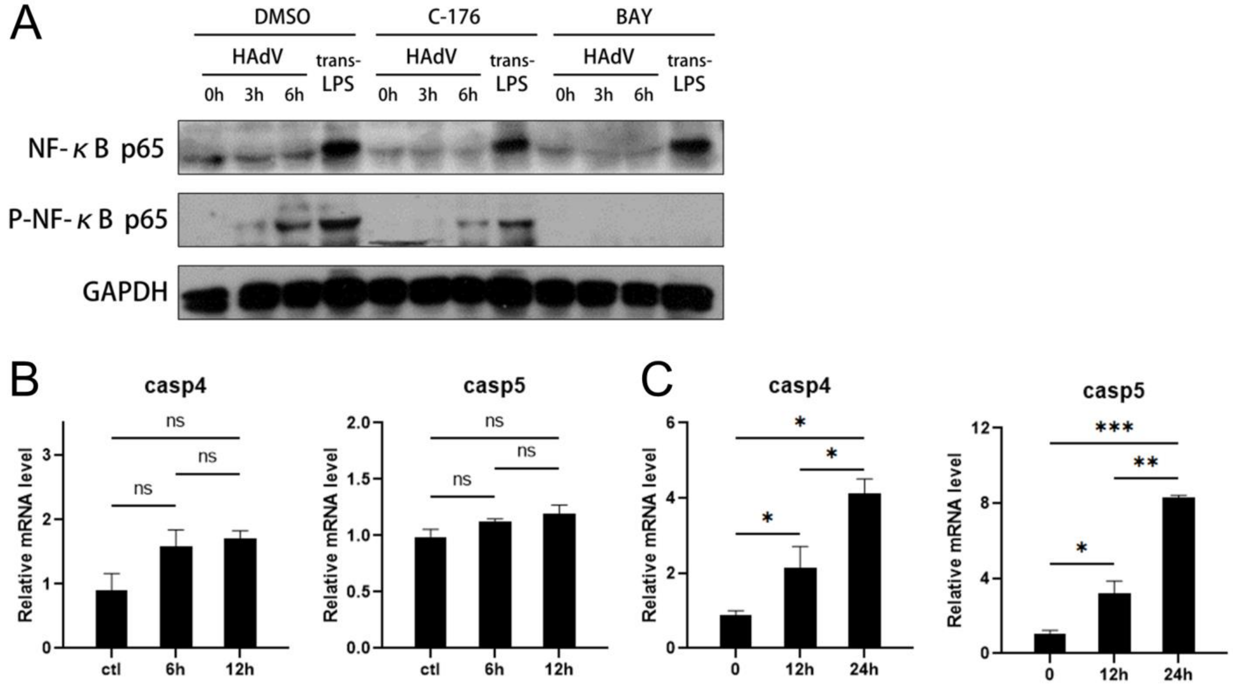

Supplementary Figure 1. HAdV infection induced NF-κB activation by TLR9. (A)The dTHP-1 cells pre-treated by either C-176 (1μM) or BAY11-7082 (10nM) vs vehicle control (DMSO) were infected with HAdV (MOI=100) for the indicated times or transfected with LPS (2.5μg/ml) for 3h. Protein levels of indicated molecular in cell lysates were tested by western blot. The mRNA levels of caspase-4 and caspase-5 responses to (B) cGAMP transfection (10μg/ml) at 0, 6, and 12h and (C) CpG ODN 2006 stimulation (10μg/ml) at 0, 12, and 24h were measured by real-time PCR. Data are representative of at least three experiments. Error bars represent the mean ± SEM. **P* value<0.05, ***P* value <0.01, ****P* value <0.001, ns. no significance.

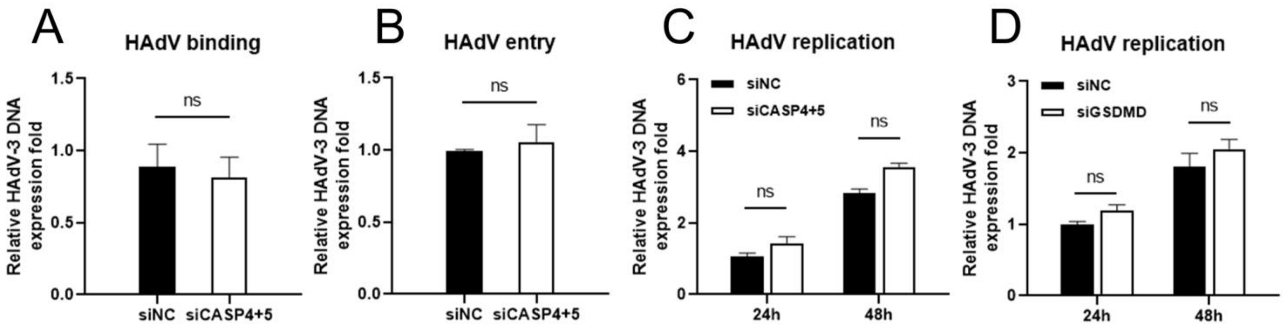

Supplementary Figure 2. Caspase-4 and caspase-5 had no effect on HAdV binding, entry and replication. (A) The dTHP-1 cells were transfected with siCASP4+5 vs siNC and then incubated with HAdV (MOI=100) in 4 ℃ for 1h to assess HAdV binding. (B) Cells were cultured in 37 ℃ for 0.5h to assess HAdV entry. (C) The siCASP4+5 or (D) siGSDMD-treated vs siNC-treated dTHP-1 cells were cultured in 37 ℃ for indicated timepoints post-infection to assess intracellular HAdV replication. HAdV DNA levels were measured by real-time PCR. Data are representative of at least three experiments. Error bars represent the mean ± SEM. ns. no significance.
