# Supplementary material for: Human adenovirus infection induces pulmonary inflammatory damage by triggering noncanonical inflammasomes activation and macrophage pyroptosis
Source: Front Immunol. 2023 Apr 18;14:1169968. doi: 10.3389/fimmu.2023.1169968 (PMC10167768; doi:10.3389/fimmu.2023.1169968)
Supplement: Supplementary file 2 [file Table_1.docx]

Supplementary Table

Human Adenovirus Infection Induces Pulmonary Inflammatory Damage by Triggering Noncanonical Inflammasomes Activation and Macrophage Pyroptosis

Lexi Li^1, 2†^, Huifeng Fan^2†^, Jinyu Zhou^3^, Xuehua Xu^2^, Diyuan Yang^2^, Minhao Wu^3^, Can Cao^3*^, Gen Lu^2, 1*^

**^†^**The authors contributed equally to this work.

*** Correspondence:**Gen Lu, M.D., Ph.D.
E-mail: [lugen5663330@sina.com](mailto:lugen5663330@sina.com)

Can Cao.
E-mail: [caoc9@mail.sysu.edu.cn](mailto:wuminhao@mail.sysu.edu.cn)

Minhao Wu, Ph.D.
E-mail: [wuminhao@mail.sysu.edu.cn](mailto:wuminhao@mail.sysu.edu.cn)

# Supplementary Table 1 Primers list

| Gene | Sequence(5'-3') | |
| --- | --- | --- |
| IFN-β | Forward | AAACTCATGAGCAGTCTGCA |
|  | Reverse | AGGAGATCTTCAGTTTCGGAGG |
| IL-1β | Forward | ATGATGGCTTATTACAGTGGCAA |
|  | Reverse | GTCGGAGATTCGTAGCTGGA |
| CASP-1 | Forward | TTTCCGCAAGGTTCGATTTTCA |
|  | Reverse | GGCATCTGCGCTCTACCATC |
| CASP-4 | Forward | ACAATGGGCTCTATCTTC |
|  | Reverse | AGTCGTTCTATGGTGGG |
| CASP-5 | Forward | CATTACGGAACTCATCACA |
|  | Reverse | TGCCAGGAAAGAGGTAG |
| β-actin | Forward | AGCGAGCATCCCCCAAAGTT |
|  | Reverse | GGGCACGAAGGCTCATCATT |
| HAdV-3 | Forward | GGGAGACAATATTACTAAAGAAGGTGTGC |
|  | Reverse | CAACTTGAGGCTCTGGCTGATA |
